# Sonochemical synthesis of heterostructured ZnO/Bi_2_O_3_ for photocatalytic desulfurization

**DOI:** 10.1038/s41598-023-46344-0

**Published:** 2023-11-08

**Authors:** Rawan M. A. Mahmoud, Fatma N. Sayed, Mohamed R. Shehata, Ahmed M. A. El Naggar, Gehad G. Mohamed, Ahmad M. Abdelaal, Asmaa S. Morshedy

**Affiliations:** 1 Chemistry Department, Faculty of Science, Cairo University, Giza, 12613 Egypt; 2 Refining Department, Egyptian Petroleum Research Institute (EPRI), 1 Ahmed El-Zomor St., Nasr City, Cairo 11727 Egypt; 3 Department of Nanoscience, Basic and Applied Sciences Institute, Egypt-Japan University of Science and Technology, New Borg El Arab, Alexandria 21934 Egypt

**Keywords:** Catalysis, Energy, Environmental chemistry, Green chemistry, Inorganic chemistry, Materials chemistry, Photochemistry

## Abstract

In this study, metal oxides nanoparticles heterogeneous photocatalysts prepared by coprecipitation and ultrasonic techniques were used for diesel desulfurization. They were characterized by scanning electron microscope, powder X-ray diffraction, energy dispersive analysis, diffused reflectance spectra, photoluminescence analysis and BET surface area. The surface area of catalyst B is larger than catalyst A confirming its higher reactivity. X-ray reflectance spectroscopy was used to analyze the sulfur contents in feed. Thiophene was used as a model fuel to evaluate the photocatalytic activity of catalysts A and B. Using the Scherrer equation, sharp and intense signals suggesting their higher degrees of crystallinity, with average crystal sizes for ZnO, Bi_2_O_3_, catalysts A and B, respectively; of 18, 14.3, 29.7, and 23.8 nm. The operational parameters of the desulfurization process were optimized and have been studied and the maximum sulfur removal was achieved via a further solvent extraction step. A diesel fuel with a 24 and 19 ppm sulfur content and hence a total sulfur removal of 94.6% and 95.7% was acquired for catalysts A and B, respectively (sulfur compounds concentration in diesel fuel feedstock was 450 ppm). These findings demonstrated that photocatalysts A and B are good and effective catalysts for desulfurization of diesel fuel.

## Introduction

The study of nanoscale materials and devices, including their design, production, characterization, and application, is known as nanotechnology. This field of research, which is a subclassification of technology in the scientific disciplines of colloidal science, biology, physics, chemistry, and others, entails the investigation of phenomena and the handling of nanoscale materials. Due to the success of nanotechnology in consumer products and other industries, nanoscale materials have the potential to benefit the environment directly by detecting, preventing, and removing pollutants^1,2^. The world is more conscious of a potential global disaster as a result of environmental pollution and energy shortages. The creation of pollution-free technology for environmental remediation and alternative clean energy sources is an urgent problem for the sustainable growth of human society. One of the most promising green energy and renewable energy project technologies is semiconductor photocatalysis because it offers a simple approach to absorbing the energy of either natural sunlight or artificial indoor lighting, which is readily available everywhere in the globe^3^.

In the area of irradiation semiconductor photochemistry, photocatalysis’s function is to start or enhance several redox (reduction and oxidation) reactions. When the input photon energy meets or exceeds the bandgap, light is absorbed and the resulting photoexcitation of electron–hole pairs occurs. When exposed to sunlight, semiconductor photocatalysts are activated, producing electrons (e^−^) with reduction capabilities in their conduction band (CB) and holes (h^+^) with oxidation capabilities in their valance band (VB)^4,5^. There has been a lot of interest recently in the creation of novel photocatalytic technology, which has a wide range of environmental applications, including the treatment of water and the cleanup of fuel oil. There are numerous advantages of using photocatalysts for environmental remediation, using solar energy to transform pollutants from complicated molecules into simple, benign compounds and prevent subsequent treatment, disposal, or the use of any expensive oxidizing chemicals^6^.

Research on ultra-low Sulphur diesel (ULSD) is crucial for both human health and environmental restoration. Fuel impurities that contain organosulfur severely pollute the environment and harm the catalysts used in gas converters for vehicle exhaust, the principal pollutant is benzothiophene and thiophene and their derivatives are sulfur-containing substances found in fuels. The creation of ultra-low sulfur diesel largely depends on eliminating components that contain sulfur^7,8^. As a result of growing environmental concerns, the removal of organosulfur compounds from fuels has received a lot of attention. Sulfur oxides, particularly SO_2_ (dominant oxide), are released into the environment when S-containing fuels are ignited, and they have the potential to harm human health and the environment. Because of the increasing restrictions, removing SCCs from fuels is attracting more and more attention today to satisfy the requirements for ultra-low Sulphur liquid fuels in terms of quality. Several methods are used for desulfurization such as hydrogenation (HDS) which is currently the industrial technique that is most often employed, and it is quite effective in removing sulfides, disulfides, and light thiophenic sulfur compounds. However, the HDS process requires operating at high pressure and temperature while using a lot of hydrogen gas. On the other hand, dibenzothiophene and other alkyl-substituted derivatives of dibenzothiophene are refractory sulfur compounds that HDS is less effective in removing, which makes this method difficult in producing ultralow sulfur fuel to meet global requirements (10 ppm). Biodesulfurization (BDS) utilizing enzymes or microorganisms as catalysts, for removing organic sulfur compounds from petroleum distillates via a unique anaerobic, damaging mechanism, application of BDS leads to microbe contamination in fuels^9,10^. Another method is adsorption desulfurization (ADS) in which the removal of sulfur compounds appears to be highly promising in terms of energy consumption compared to the hydrodesulfurization process since adsorption can be completed at low temperatures and pressures and the sulfur in fuels can be reduced to a very low level^11,12^. Oxidative desulfurization (ODS)^13,14^, and finally photocatalysis method. This method considered the most effective and conventional study so far demonstrated that deep desulfurization of transportation fuel by oxidative desulfurization (PODS) can be carried out with good product selectivity at room temperature and atmospheric pressure^15^. The use of ultrasound can improve mass transfer in heterogeneous systems and enhance kinetics, which can accelerate the reaction rate due to high cavitation activity, based on photocatalytic oxidative desulfurization^16^.

In this research, we selected a coupling of appropriate semiconductors that are inexpensive, effective in visible light, and have good charge separation, the semiconductors used are zinc oxide (ZnO) which is one of the most popular semiconductor photocatalysts because of their high stability, low price, absence of toxicity it also possess greater photocatalytic potential, although the efficiency of ZnO as photocatalyst it is active only in UV radiation and fast (e^−^,h^+^) recombination, so using a coupled semiconductors is one of the most effective techniques to improve electron–hole separation. (Bi_2_O_3_) is a significant p-type semiconductor with four major crystallographic polymorphs, which is an abundant material, and has a band gap in visible range (2.8 eV). This couple was prepared using a chemical precipitation procedure with ultrasonic assistance, and the resulting sample was used to develop a low-sulfur model diesel^17–20^.

## Experimental

### Materials

Anhydrous zinc chloride (ZnCl_2_), anhydrous bismuth nitrate Bi(NO_3_)_3_, ammonium hydroxide solution, absolute ethanol with purity 99% which all provided from Sigma-Aldrich Chemie GmbHmodel diesel fuel fraction purchased from Egyptian Petroleum Research Institute (EPRI) with concentration 450 ppm, distilled water, acetonitrile, Linear halogen lamp 500 Watt (LHL)was purchased from Abo-Symbol Company (Cairo, Egypt as a representative of Light Sources), reflux condenser, round flask, stirrer bar.

### Synthesis procedures

ZnO NPs and Bi_2_O_3_ NPs were produced by preparing 0.4 M solution of anhydrous ZnCl_2_ and Bi (NO_3_)_3_ salts_,_ respectively, which then precipitated by diluted ammonium hydroxide solution (1:1 v/v) gradually until reaching to pH (10.5–11), the precipitate were then filtrated and washed then calcinated at 500 °C for 4 h.

ZnO@Bi_2_O_3_ composites (catalyst A) is a mixture of photocatalytic semiconductors prepared by the chemical precipitation (co-precipitation) method as described here. A solution of 0.4 M of Bi(NO_3_)_3_ was mixed with 0.4 M solution of ZnCl_2_ by ratio (20:80% v/v). The obtained mixture was precipitated by adding diluted ammonia solution (1:1 v/v) gradually with continuous stirring until reached pH = 10.5–11. The obtained solid product was collected by filtration and washed with distilled water. Then, it was left to dry at room temperature and finally calcined at 500 °C for 4 h.

ZnO@Bi_2_O_3_ (catalyst B) was prepared by mixing Bi_2_O_3_ with a 0.4 M solution of ZnCl_2_(20:80 w/w) and precipitated using ammonium hydroxide till reaching pH = 11. The precipitate was collected and left for drying. After that, the composite was subjected to ultrasonic oscillation with absolute ethanol. The ultrasonic vibration was afterward stopped, the precipitated was then filtrated and washed, and the obtained catalyst was calcinated at 500 °C for 4 h^16,20,21^.

### Procedure for desulfurization

The produced semiconductor photocatalysts were transported to the sulfur removal step once their full properties were evaluated. All tests were conducted in a wooden box that contained a round flask of 50 ml, water-cooling system (reflux) was used to keep the temperature stable during the photoreaction. The desulfurization procedures began by charging the reaction vessel that contains semiconductors photocatalysts, feedstock and magnet. To begin the procedure, the entire system was exposed to the irradiation source which namely a linear halogen with a power of 500W, was used. Supplementary Figure S1 depicts the setup utilized in the sulfur removal procedure. This design was created by the authors of this study. Different factors may influence the degree of sulfur removal from diesel fuel by photocatalysis. Several variables were investigated in this study, including the influence of the catalyst dosage, reaction time, different volumes of oxidizing agent H_2_O_2,_ and solvent ratio, these ranges and conditions are chosen according to the literature survey and according to our experience with such work. Lot of trials to choose the best range and conditions for the work^22^. At the end of this stage, the optimal conditions identified were applied to the desulfurization of diesel fuel while exposed to sunlight. Following this process, the resulting product was extracted at the indicated optimal sulfur/feed ratio^22^. The product was determined using the XRF instrument.

### Instrumentation

Powder X-ray diffraction XRD (model Brucker D8 discover) in Egyptian Nanotechnology Center, Cairo University. The scanning electron microscope SEM model (Leo Supra 55) in the Egyptian nanotechnology center, Cairo University. BET surface area model (Nova touch xl2) was conducted, and Differential reflectance spectroscopy (DRS) model (Jasco-V-570 Japan); in the range of wavelength of 200–2000 nm at room temperature in Metallurgical Development Research Center, Egypt. The photoluminescence (PL) spectra were collected using the Spectrofluorophotometer model SHIMADZU, RF-5301, Egyptian Petroleum Research Institute. The X-ray reflectance spectroscopy (XRF) model (EDXRF spectroscan (SL) was done at the Egyptian Petroleum Research Institute. Magnetic stirrer and pH meter.

## Results and discussion

This chapter includes the full characterization of the prepared composites of (ZnO@Bi_2_O_3_) by two different methods, it also includes the photocatalytic activity of the composites in the desulfurization of diesel fuel that was presented previously.

### XRD analysis

**Figure 1:**
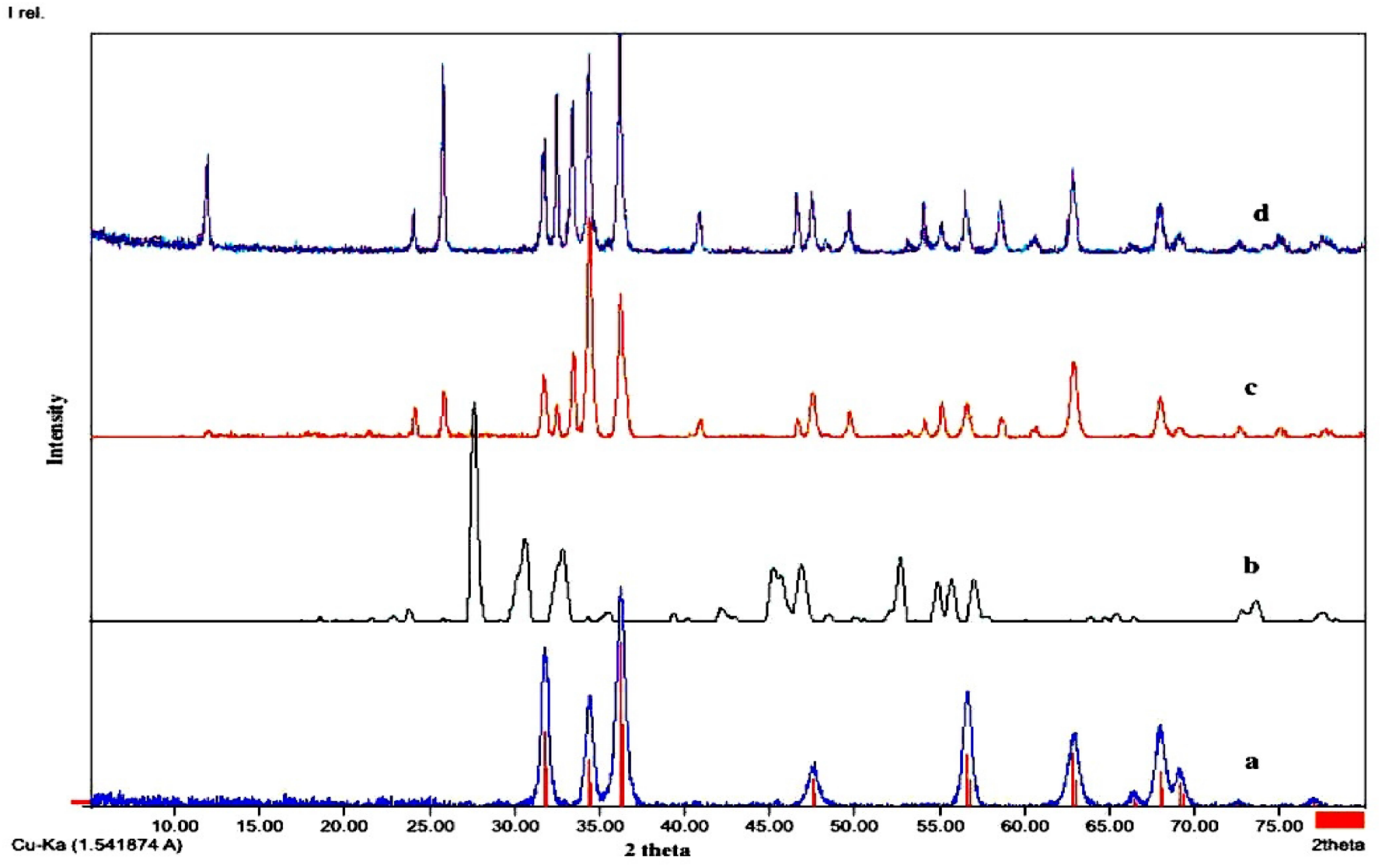
X-ray diffraction patterns of (**a**) ZnO, (**b**) Bi_2_O_3_, (**c**) catalyst A and (**d**) catalyst B.

The structural characteristics of the prepared nanostructures (ZnO and Bi_2_O_3_) and photocatalysts (A and B) are identified through the exhibited XRD patterns as illustrated in Fig. 1. The displayed XRD signals for pure ZnO (Fig. 1a) revealed the presence of hexagonal phase based on observation of indicative diffraction peaks at 31.77°, 34.42°, 36.25°, 47.54°, 56.40°, 62.68° and 67.72° These signals are respectively corresponding to the following lattices planes: (100), (002), (101), (102), (110), (103) and (112) according to the (COD 2107059) and space group: P63. The XRD spectrum of the Bi_2_O_3_ semiconductor (Fig. 1b), which was prepared at the same conditions as ZnO, showed indicative peaks at 27.42°, and 33.31°. The observed XRD peaks correspond respectively to (120) and (220) as lattice planes, in a match to space group: P 1 21/c and the diffraction phase: (COD 1010004)^17,21^. For the two composite photocatalysts A and B, indicative peaks for both ZnO and Bi_2_O_3_ could be simultaneously noticed, as shown in the provided XRD spectra in Figs. 1c and d. The detection of these peaks in the illustrated structures could verify the successful preparation of the designated composites. Nevertheless, the noticed peaks in these two composites are of less intensity than in parent metal oxides and are a bit shifted than their original positions. These differences are explained due to the incorporation and dispersion of the two metal oxides within and in between each other lattices as well as the accumulation of some of their particles. On the other hand, the four investigated photocatalysts revealed sharp and intense signals indicating their increased degrees of crystallinity. The average crystal sizes are found to be 18, 14.3, 29.7, and 23.8 nm for ZnO, Bi_2_O_3_, and catalysts A and B, calculated from the Scherrer equation as shown below.

$$Dc = \frac{{{\text{K}}\uplambda }}{\beta /2cos\theta }$$

where Dc is the crystalline diameter in nm; K is the Scherrer constant; λ is the wavelength of the X-ray radiation; β is the FWHM of the diffraction peak; and θ is the peak diffraction angle size. They noticed bigger mean crystal sizes for catalysts A and B confirming the interferences of both ZnO and Bi_2_O_3_ oxides during the preparation of these composites. Because of the higher crystallinity and the average nano-crystal size that appeared in this work, the photocatalytic properties and decomposition of the sulfur compound were enhanced^22–25^.

### BET surface area analysis

The surface characteristics of the two blank metal oxides and their sub-driven catalysts A and B are illustrated in Table 1. Additionally, N_2_ adsorption–desorption isotherms of the four synthesized structures are presented in Figure S2. The prepared structures showed reasonable specific surface area values, as detected from the multi-point BET analysis. The noted surface area values are a strong match with the calculated average crystal sizes from XRD analysis where Bi_2_O_3_ showed the highest value of the surface area and smallest crystal size. The increased S_BET_ of this structure over the other three photocatalysts is explained by having the largest total pore volume and smallest pore diameter. On the other hand, the observation of a slightly bigger average pore diameter for catalysts A and B than parent metal oxides refer to the overlap and incorporation of the ZnO and Bi_2_O_3_ lattices, as previously stated via XRD analysis. This finding could further confirm their reduced specific surface area values compared to blank metal oxides due to the formation of some agglomerates of the metal oxide particles during the composite’s preparation. In addition, the surface area of catalyst B is larger than A which means higher reactivity for catalyst B due to the greater surface area, the greater the number of active photocatalytic sites, the higher the adsorption capacity for pollutants on the photocatalyst surface, and the greater the photocatalytic activity. About the provided isotherms in Figure S2, the two metal oxides and sub-prepared composites had demonstrated materials of type IV, according to the IUPAC classification. Hysteresis loops of type H3 referring to materials with a narrow mesoporous nature could be noted for the four displayed isotherms in Figure S2^23,26–28^.

**Table 1:** Surface parameters of ZnO, Bi_2_O_3_ and their subsequently produced composites.

Sample	BET surface area (m^2^/g)	Total pore volume (cm^3^/g)	Average pore size (nm)
ZnO	64.24	0.2460	8.20
Bi_2_O_3_	74.16	0.2873	7.58
Catalyst A	47.50	0.1840	8.50
Catalyst B	55.484	0.2124	8.21

### Morphological structure

**Figure 2:**
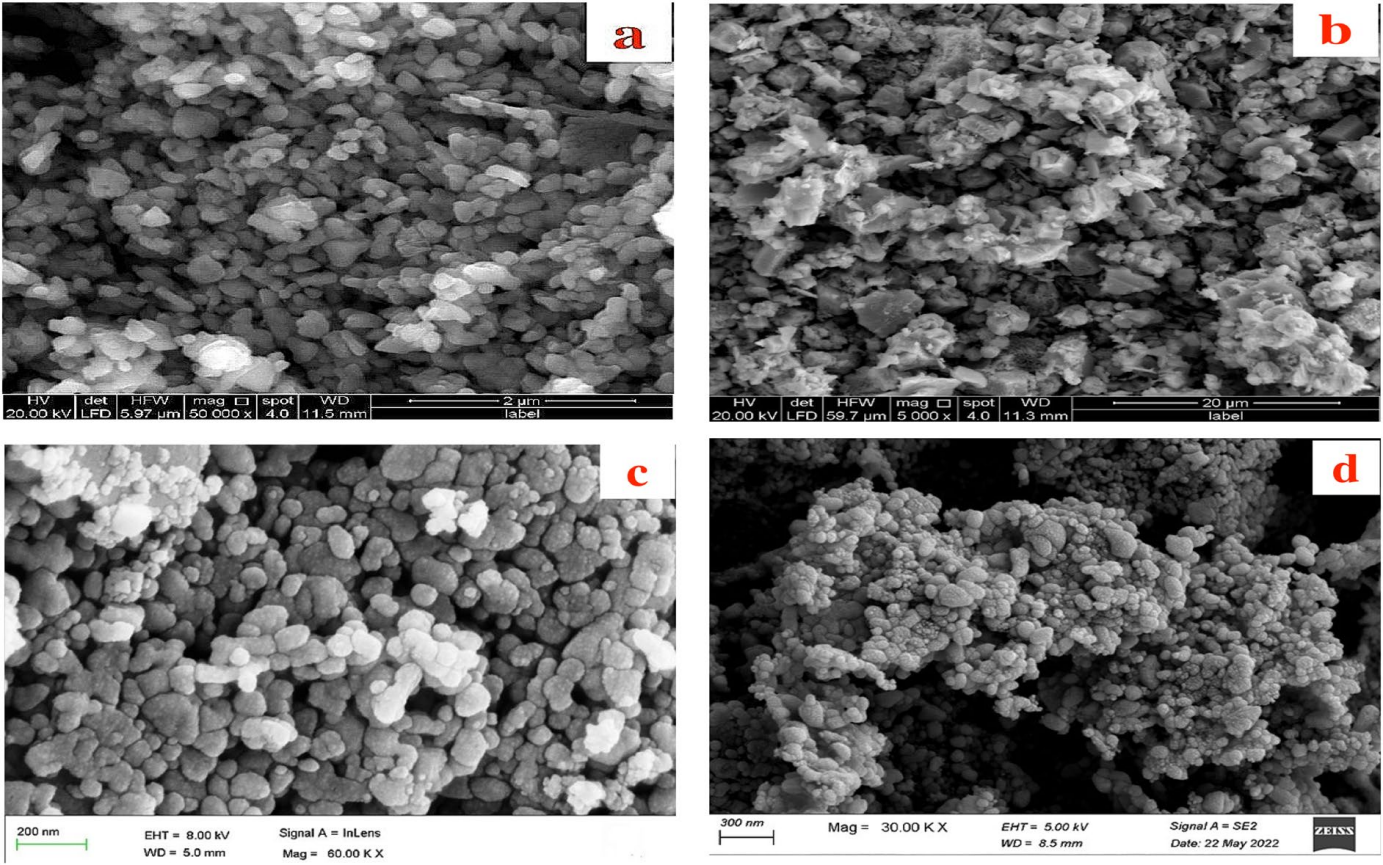
Surface morphology (by SEM) of (**a**) ZnO, (**b**) Bi_2_O_3_, (**c**) catalyst A and (**d**) catalyst B.

The morphologies of ZnO, Bi_2_O_3_, and catalysts A and B are illustrated via the displayed SEM images in Fig. 2. ZnO showed quite uniformly distributed granular particles have tetragonal shapes that are ordered together to provide a surface that has, to some extent, a smooth appearance (Fig. 2a). Several ores have different sizes could be noticed as embedded in between the ZnO particles which exhibited Sizes as distributed from 12 to 39 nm. Figure 2b discusses the morphology of Bi_2_O_3_ which revealed particles of uniform distribution that provide a strongly wavy nature due to the observation of popcorn-like particles. Unlike ZnO, quite increased porous morphology could be noted for Bi_2_O_3_ which is in agreement with the previously provided data from surface area analysis. The displayed SEM image showed particles have sizes in the range of 10–32 nm. On the other hand, the incorporation of ZnO and Bi_2_O_3_ particles together in the same structures (catalysts A and B) could have obvious changes in their morphological features. For catalyst A, (Fig. 2c), uniform morphology through the detection of oval-shaped particles has sizes ranging between 20 and 46 nm could be noticed. Explicit non-smooth morphology is noted for this structure which is due to the accumulation of some particles through the formation of such composite structure. Figure 2d presents the surface appearance of catalyst B which has extremely wavy and layered morphology. The different morphology of catalyst B compared to the other three structures can be referred to the effect of using ultrasonic homogenizing during its preparation stage that could lead to high dispersion of metal oxide particles within each other and leading to enhancement of the photocatalysis performance, reducing reaction time, avoiding the use of extreme conditions and improving the properties of the photocatalytic material. This in turn could result in a stage of re-building and re-construction stage producing particles of different shapes. Particularly, triangular, tetrahedral, and pentagon-shaped particles sized from 18 to 39 nm could be detected.

**Figure 3:**
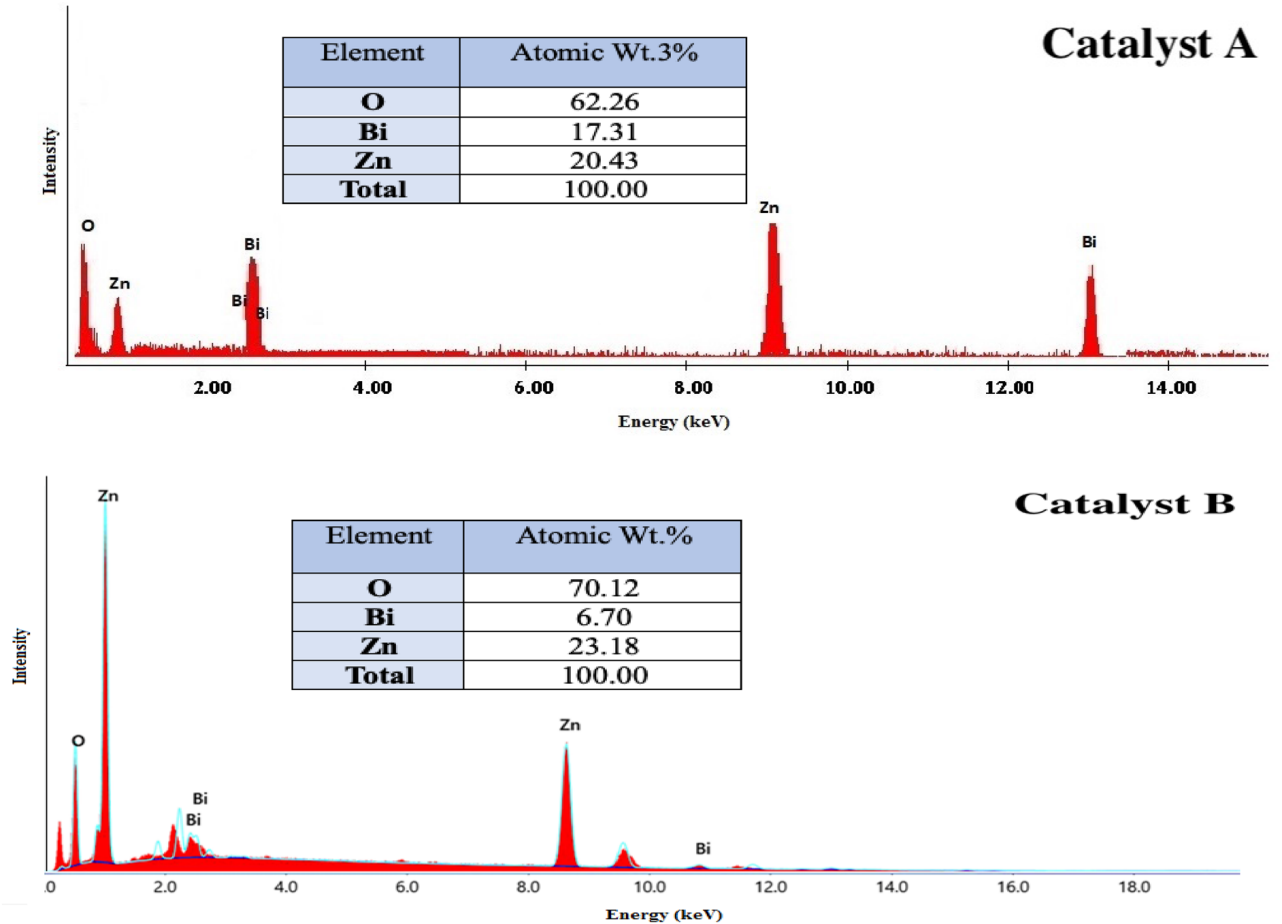
EDX spectra of (**a**) catalyst A and (**b**) catalyst B.

The chemical compositions of these two composites (catalysts A and B) are further verified through the provided EDX spectra in Fig. 3. Sharp intense peaks indicative of Zn, O, and Bi elements are observed for both structures which confirm their chemical formulations as mixed oxides. Nevertheless, the intensities of Zn metal in catalyst B are much higher than that in catalyst A which was compromised by an obvious reduction in the intensity of its Bi signal. These observations are attributed to the incorporation of the two metal oxide lattices and mutual rebuild, as previously suggested through SEM, due to the effect of ultrasonic waves. This could in turn potentially release Zn atoms to be superficial in the produced composite while Bi ones could be internally embedded in the bulk of the catalyst structure. On the other side, no other signals, a part of the previously stated three elements, were noticed through the given EDX spectra for both catalysts A and B. This notice could strongly emphasize the formation of the two composites in a state of high purity^24,27^.

### Optical characteristics

**Figure 4:**
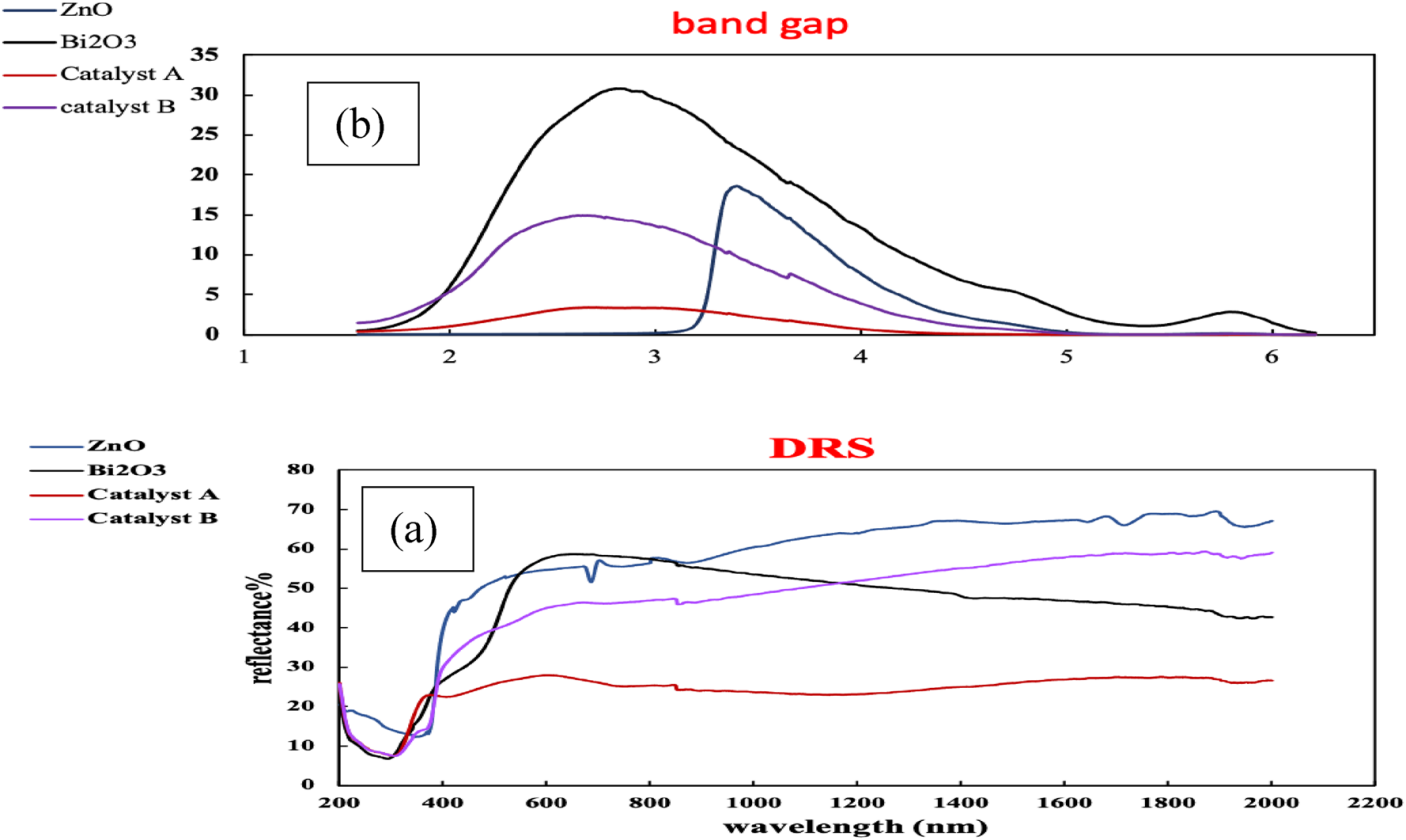
(a) DRS and (b) bandgap of ZnO, Bi_2_O_3_, catalyst A and catalyst B.

**Figure 5:**
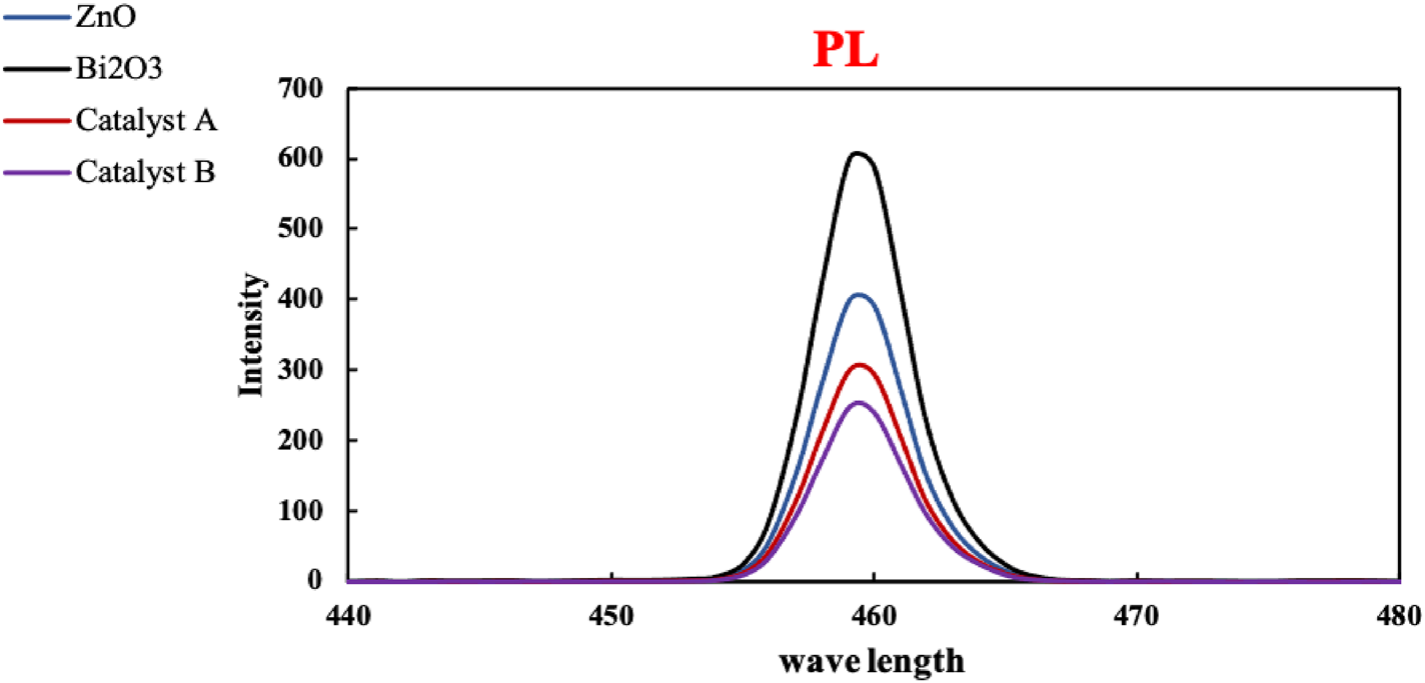
PL curves of the as-prepared parent metals oxides, catalysts A and B.

The photo-optical properties of the produced blank metals oxides and their subsequently generated composites ZnO@Bi_2_O_3_ are investigated through the displayed UV–visible diffuse reflectance spectroscopic (DRS) spectra and photoluminescence (PL) curves in Figs. 4 and 5 respectively. The UV–visible DRS spectra (Fig. 4) showed that ZnO has an absorption peak relatively narrow to 350 nm with a band gap value between (3–3.5 eV) reflecting its effective optical photo-activity in the region of UV radiation. On the other hand, Bi_2_O_3_ and the two composite structures (catalysts A and B) were found to be actively sensitive in the region of visible light irradiation owing to the observation of absorption bands in the region between 450 and 520 nm. These absorption peaks correspond to band gap values of a quite narrow range; specifically, between 1.6 and 2.5 eV. It could be seen that the incorporation of both ZnO and Bi_2_O_3_ in the same structure (catalysts A and B) had in turn resulted in shifting the band gap of ZnO from UV to visible light region. This band gap shift is attributed to the interference between the present electrons in the outermost shells (energy level of valence band) in both metals’ oxides. This interaction consequently stimulates the movement of these electrons to upper sub-energy levels. Hence, less quantum of energy is needed to transfer the electrons from the valence to the conduction band. Therefore, the values of the energy band gap for both catalysts A and B are reduced from nearly 3.3 to below 2.5 eV.

The optical characteristics of the introduced structures are further investigated through the provided PL charts in Fig. 5. It can be seen that both ZnO and Bi_2_O_3_ showed curves with quite high intensities which reflects the rapid recombination of their electron–hole pairs during light illumination. On the other side, catalyst B has the lowest PL intensity, hence as a result, it has the maximum delay of electron–hole recombination during the excitation by light radiation. Therefore, catalyst B, among the four structures, is potentially of the most distinctive performance based on the rule stating that the lower the intensity of the PL curve, the higher the photo-activity. About Fig. 5, the order of PL intensities is as follows: catalyst B < catalyst A < ZnO < Bi_2_O_3_. Thus, it can be concluded that both catalysts B and A are expected to display outstanding photocatalytic activities since they have the slowest rates of recombining the electron–hole pairs. This delay in pairs recombination is explained due to the change of charge transfer pathway due to the incorporation of both ZnO and Bi_2_O_3_ in one composite. Particularly, the excited electrons of ZnO in its conduction band are encountering a stage of relaxation on their way back to the ground state. These electrons first travel to the conduction band of Bi_2_O_3_, then they continue their way to the valence band of ZnO^29^. Therefore, an obvious delay in the recombination between the excited electrons and the holes they left behind at the catalyst surface could take place. On the other side, the lower intensity of the PL curve for Catalyst B than that of Catalyst A can be attributed to the different methods of preparing each of them. Specifically, the use of ultrasonic waves for preparing catalyst B could result in collapse and re-construction for the particles of metal oxides leading to stronger inferences between their electron clouds. This overlap of electrons could restrict the charge transfer during light illumination, thus, a delay in e-hole pairs recombination is undertaken^24,30,31^.

### Photocatalytic activity in sulfur removal

After complete characterization of the as-synthesized catalysts and demonstration of their various photo-optical properties, both catalysts A and B were selected to perform the process of diesel fuel desulfurization since they revealed outstanding features, in comparison to the parent metals oxides. The efficiencies of these two structures in the removal of sulfur compounds were investigated in the presence of visible light and under the influence of different operating parameters^23^.

#### Effect of catalysts dose

**Figure 6:**
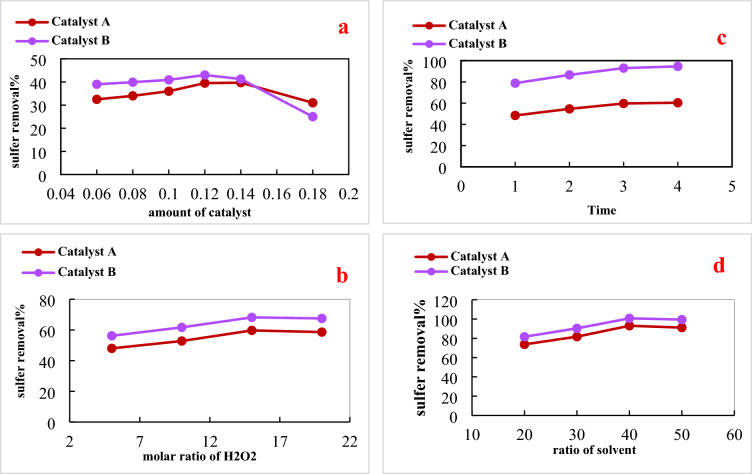
Photocatalytic activity under different conditions (**a**) catalyst dosage, (**b**) effect of H_2_O_2_, (**c**) effect of time, (**d**) solvent ratio for catalyst A and (**e**) solvent ratio for catalyst B.

In this stage, the effect of using different amounts of the photocatalysts on the process of diesel fuel desulfurization was tested. Practically, the sulfur removal experiments were carried out at an operating time of 3 h using catalyst dosages of 0.06, 0.08, 0.1, 0.12, 0.14, and 0.18 g while a fixed volume of feedstock (20 mL) was introduced for the reaction. Figure 6a illustrates the desulfurization percentages versus the change of catalysts dose. For both photocatalysts, it can be observed that increasing the amount of catalyst up to 0.12 g could continuously improve the efficiency of the sulfur removal process. This increment in desulfurization percentages is attributed to the increase in the number of active sites by an increase in catalyst dose. The use of photocatalysts amount of more than 0.12 g could be accompanied by decreases in sulfur removal percentages. This percentage decline is explained due to light scattering by increasing the number of photocatalysts particles. Therefore, the photo-activities of both catalysts are reduced^22,32,33^.

#### Impact of oxidizing agent (H_2_O_2_) content

By the end of the prior stage, a catalyst dose of 0.12 g was chosen as the optimum to be employed in the current stage through which the influence of H_2_O_2_ to feed ratio on the level of desulfurization was studied. H_2_O_2_ is used to increase desulfurization reactivity and avoid ODS processes from being inhibited^13^, under the effect of light radiation, hydrogen peroxide forms highly reactive hydroxyl radicals (OH^•^) that can oxidize organic sulfur species producing sulfoxides and sulfones, as provided in the following Eqs. (1–5):

1 $${\text{H}}_{{2}} {\text{O}}_{{2}} + {\text{hv}} \to {\text{2OH}}^{ \cdot }$$

2 $${\text{2H}}_{{2}} {\text{O}}_{{2}} \to {\text{2H}}_{{2}} {\text{O}} + {\text{O}}_{{2}}$$

3 $${\text{e}}^{ - } + {\text{ H}}_{{2}} {\text{O}}_{{2}} \to {\text{OH}}^{ \cdot } + {\text{OH}}^{ - }$$

4 $${\text{h}}^{ + } + {\text{OH}}^{ - } \to {\text{OH}}^{ \cdot }$$

5 $${\text{e}}^{ - } + {\text{O}}_{{2}} \to {\text{O}}_{{2}}^{. - }$$

Different volumes of H_2_O_2_; namely, 5, 10, 15, and 20 mL were utilized in this step of investigation while applying 3 h as processing time and using a volume of feed equal to 20 ml. The impact of oxidizing agent increase on the percentages of sulfur removal is illustrated in Fig. 6b. The sulfur removal was increased by the elevation of the hydrogen peroxide volume up to 15 ml. The noticed inflations in desulfurization degrees are attributed to the increase in the number of OH radicals that could consequently increase the probability of oxidizing the present sulfur compounds in the diesel fuel fraction into sulfones. These oxidized components could be then easily eliminated from the diesel fuel via adsorption on the catalyst or by the existing water molecules in processing media, due to H_2_O_2_ decomposition, which acts as a solvent. The use of an excess amount of oxidizing agent (more than 15 mL) could have a negative impact on the desulfurization percentage. The detected decrease in desulfurization activity can be explained by the increased absorption of light radiations by increasing the amount of H_2_O_2_ in the reaction vessel. Therefore, limited photo-activity could be attained by the particles of the catalysis, leading to the decrease of sulfur compounds removal^18,34,35^.

#### Effect of reaction time

By the completion of the former stages, the catalyst does of 0.12 g and 15 mL of H_2_O_2_ was picked as the optimum conditions to be employed during the current desulfurization study. In this stage, the effect of using different operating times on the quality of the desulfurization process is checked. The collected desulfurization percentages at a time range of 1–4 h, with a time increase interval of 1 h, are presented in Fig. 6c. The desulfurization percentages were increased by lasting the process for a longer time to reach the maximum at 3 h. This observation refers to the increased interactions between the catalyst’s particles and diesel fuel molecules by increasing the operational times. The following increase of the reaction time to 4 h is associated with observing a nearly steady state of sulfur removal exploits. This finding could be explained by covering the catalyst’s surfaces with mono-layers of adsorbed sulfur compounds. This coverage of surfaces could in turn limit the performed photocatalytic activities by the two employed catalysts. Thus, applying an operational time of 3 h was found to be the optimum to achieve successful desulfurization activities by the two presented catalysts in the current study.

Along the three previous stages, it could be noticed that catalyst B always has a higher desulfurization percentage than catalyst A. This observation refers to the better textural and optical properties of catalyst B than catalyst A. Particularly, catalyst B showed slower e–h pairs recombination, smaller band gap value, and bigger specific surface area value than catalyst A. Therefore, increased photo-activity is strongly expected by catalyst B over catalyst A, as displayed in Figs. 6a–c^16,32^.

#### Effect of solvent extraction process

At the end of studying the affecting parameters on the photocatalytic process and optimum conditions were optimized, another complementary stage was carried out to produce a high grade of environmentally friendly diesel fuel. In the current stage, the produced diesel fuel, at optimum conditions from previous stages, was forwarded for a solvent extraction step using acetonitrile. The selection of such solvent is based on its wide use as a safe and effective reagent to remove sulfur-containing compounds. Different solvent-to-feed ratios (S/F); namely, 2:1, 3:1, 4:1, and 5:1 was employed, and their corresponding desulfurization percentages are introduced in Fig. 6d. It could be seen that the increase of S/F up to 4:1 was joined by a continuous increment in desulfurization activity. This enhancement in sulfur compound removal is explained by increasing the solvent power by increasing the amount during the extraction process. Thus, increased amounts of the oxidized sulfur compounds could be eliminated. At S/F equal 4:1 sulfur removal percentage of 94.6 and 95.7% could be achieved by catalysts A and B respectively.

The use of S/F beyond the ratio of 1:4 was accompanied by decreases in the sulfur removal percentages. This decline in the desulfurization activity is referred to as the extensive increase in the solvent power with an obvious loss for a part of its selectivity. Therefore, the focus of solvent to particularly remove sulfur compounds could be reduced, hence overall desulfurization activities were minimized^36,37^.

### Catalyst regeneration

Recovery and regeneration of spent catalysts that are released after use in certain applications is a crucial stage in terms of adding good economic feasibility in a designated process. In agreement with that scope, residuals of the catalysts that were collected after completing the catalytic photochemical desulfurization, under optimal conditions, had received recycling via the following consequence. The spent catalysts were first soaked for 2 h in a mixture made of equal masses of benzene and methanol. Then, they were washed with double distilled water, under vigorous stirring, three times. At last, the washed catalysts were dried in an electric oven at 100 ºC for 3 h. The recovered catalysts were subsequently introduced for the desulfurization of a fresh diesel fuel sample at the picked optimum conditions from the former stages. The sequence of catalyst regeneration and reuse in the sulfur removal process was repeated five times in total. A nearly constant desulfurization percentage could be demonstrated by the two structures (catalysts A and B) over the first four cycles of regeneration. Slight decreases in the desulfurization performances of the two catalysts were noticed in the fifth turn of catalysts recovery. This decline can be explained due to the presence of some sulfur compounds, as strongly attached (embedded) within the catalyst’s structures, that could not be released through the regeneration steps, the desulfurization percentages for each cycle are presented in Table 2^22,38,39^.

**Table 2:** Desulfurization percentages for each cycle.

Catalyst	Trials					Catalyst	Trials				
Catalyst A	1st	2nd	3rd	4th	5th	Catalyst B	1st	2nd	3rd	4th	5th
Desulfurization percent%	94.60%	94.60%	94.50%	94.46%	94.40%	Desulfurization percent%	95.70%	95.69%	95.67%	95.65%	95.65%

### Photocatalytic mechanism

**Figure 7:**
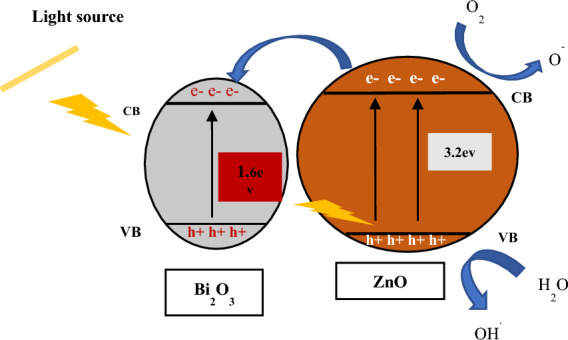
Schematic diagram of reaction mechanism of sulfur compounds removal.

The photocatalytic mechanism by the introduced catalysts in the current study is presented schematically in Fig. 7. It is obvious that by subjecting visible light on the composites, the electrons in VB of ZnO are transported to its CB resulting in an e^-^ in CB in which the reduction step occurs and h^+^ is formed in VB that executes the oxidation step. The excited electrons are then relaxed to the CB sites of Bi_2_O_3_, which delays e/h^+^ recombination in ZnO. During the excitation, the produced electrons reduced H_2_O_2_ molecules and created OH^·^ radicals which further increased the oxidation/reduction processes. The generated radicals could oxidize the sulfur compounds, changing their S content to sulfone groups (oxidized form) which could then be easily removed by using solvent at a later stage. Comparing ZnO and Bi_2_O_3_ alone with the combination of ZnO/Bi_2_O_3_ we found that the composite enhanced the photocatalytic and mineralization under visible light. This is due to a synergistic effect in which Bi_2_O_3_ extends the film’s absorption range to the visible region and proper alignment of the electronic band edges induces charge separation, resulting in a decrease in recombination rate^18^.

### Comparability of photocatalytic performance

After exploring the photo-activities of the presented structures in this study toward the desulfurization process, it was necessary to compare their performances with other photocatalysts that were previously published and available through a literature survey (Supplementary Table S1). Accordingly, the desulfurization efficiencies of (ZnO@Bi_2_O_3_) composites are found to be in a good range when compared to other structures, taking into account that real diesel fuel fraction was utilized in this study while other catalysts were mostly employed for model diesel fuel. Therefore, the high feasibility of the presented study and the good reliability of the introduced catalysts to perform efficient desulfurization of petroleum diesel fuels can be proved. Additionally, these catalysts can provide economic effectiveness through processing plants in terms of their applicability in visible light, fabrication low cost, and attainment of high desulfurization efficiency.

## Conclusion

Four nanostructured metal oxide-based materials were prepared in this study via different methodologies. Specifically, ZnO and Bi_2_O_3_ were prepared via the chemical precipitation method. Two composites made of these two metal oxides were subsequently synthesized by different routes, however, they both hafve the same chemical composition. The first one was obtained via the chemical Co-precipitation method while the second was produced by suspension of ZnO and Bi_2_O_3_ particles in ethanol under the effect of ultrasonic waves. The structural characteristics of the four materials were confirmed via XRD analysis which revealed their high degrees of crystallinity. The chemical composition and complete purity of these four structures were further acquired and verified by EDX analysis. The surface properties of the four materials were found to be strongly affected by the method of preparation, however, they are all mesoporous materials of type IV. Moreover, the morphologies of the four structures were influenced either by composition change or utilization of different methods of preparations. Nevertheless, all of them displayed quite porous nature and mostly crystal-shaped particles which is in agreement with both structural and surface features. On the other side, the two composite structures could present outstanding optical characteristics over the blank metal’s oxides. Thus, they were selected to perform the designated photocatalytic desulfurization process. The removal efficiency of total-sulfur compounds was studied using an oxidative-photocatalytic route under the effect of visible light irradiation. The results demonstrated that the elimination of sulfur compounds from diesel fuel was a feasible technique. The desulfurization process was found to be substantially more successful in the presence of H_2_O_2_ as an oxidizing agent. In addition, reaction time and catalyst dosage had significant impacts on the photocatalytic process. Desulfurization of diesel fuel (450 ppm) was carried out under visible light followed by solvent extraction with acetonitrile. Diesel fuel has a low sulfur content of 19 ppm achieving a removal percentage of 95.7% could be produced by catalyst B. Moreover, a sulfur removal percentage of 94.6% could be produced by catalyst A producing a diesel fuel that has a sulfur content of 24 ppm. The catalysts can be used recycled and reproducible results were obtained. Finally, the scientific reason for this research is to reduce air pollution by lowering toxic gas emissions (such as sulfur oxides) and other polluted materials, thereby preventing the formation of SO_2_ after combustion, and avoiding catalyst poisoning in subsequent processing steps. In addition to that, protect human health and decrease the problem of global warming.

### Supplementary Information

Supplementary Information.

## Data Availability

The datasets used and/or analyzed during the current study are available from the corresponding author on reasonable request.
