# Supplementary material for: Sonochemical synthesis of heterostructured ZnO/Bi2O3 for photocatalytic desulfurization
Source: Sci Rep. 2023 Nov 8;13:19391. doi: 10.1038/s41598-023-46344-0 (PMC10632363; doi:10.1038/s41598-023-46344-0)
Supplement: Supplementary file 1 — Supplementary Information. [file 41598_2023_46344_MOESM1_ESM.pdf]

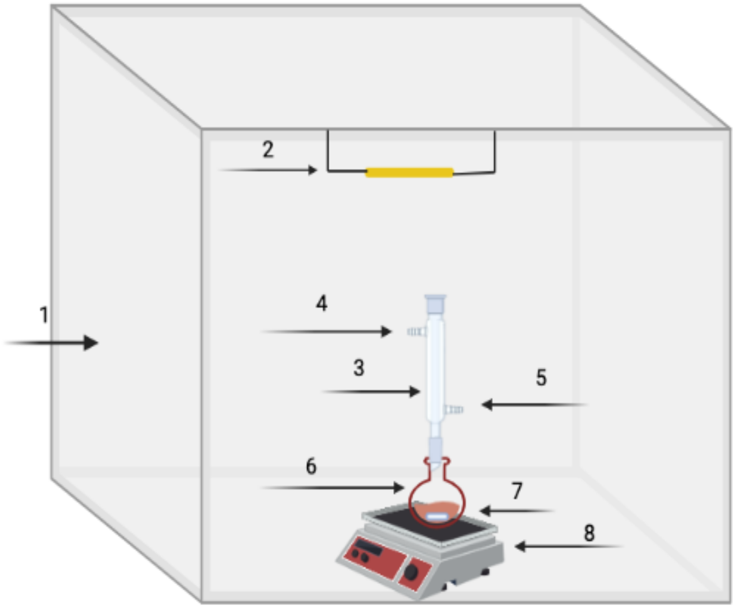

Figure S1. Photocatalytic desulfurization system. (1) Woody box, (2) linear halogen lamp, (3) glass reflux, (4) water outlet, (5) water inlet, (6) round flask containing Diesel feed,(7) stirrer bar(magnet),(8) magnetic stirrer.

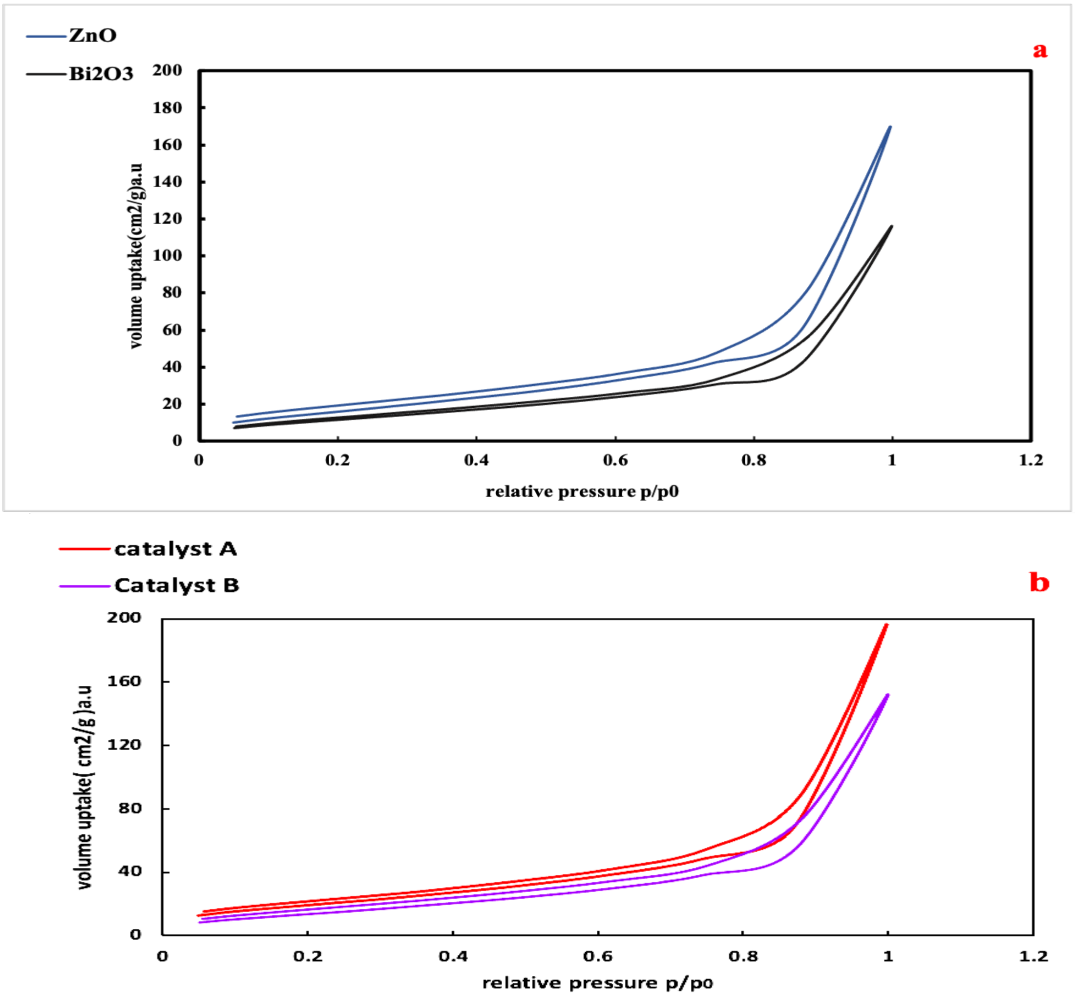

Figure S2. N_2 adsorption-desorption isotherm of (**a**) ZnO,(**b**) Bi_2O_3, (**c**) catalyst A,(**d**) catalyst B

Table S1. Comparison of previous articles with this research in the field of photocatalytic desulfurization

photocatalyst	Conditions	Sulfur removal %	References
AgI/Bi_2O_3	Model fuel, 400 W Osram visible lamp	93%	[1]
ZnO/TiO_2-SiO_2	DBT, 300W xenon lamp	97%	[2]
ZnO NPs	Model oil, UV light irradiation	97%	[3]
Mn-doped ZnO	DBT(dibenzothiophene) ($C_{12}H_8S$)(TCI:Tokyo Chemical Industry) as a standard sample of fuel and low aromatic white spirit (L.A.W.S) (AW 402) as a real sample of fuel ,visible-light irradiation(200W) tungsten	95.9%<br>94.9%	[4]
ZIF-8@Cu/Ni/ZnO@CNTs	Coal and commercial fuels, Ultra violet (UV)-Cutoff filter of 420 nm, which cutoff wavelength below 420 nm.	97%For coal, 100% For commercial fuel	[5]
BiVO4@HKUST-1 composite	model fuel containing 100 ppm, A LED lamp (40 W, 700 nm > λ > 500 nm) was used as a light source,	95%	[6]

Cu-$BiVO_4$	Model oil, metal halide lamp irradiation visible region	90%	[7]
ZnO@Bi_2O_3	Diesel fuel 450 ppm, LHL 500W		This work

References

1. Mousavi-Kamazani, M., Ghodrati, M. and Rahmatolahzadeh, R. Fabrication of Z-scheme flowerlikeAgI/Bi2O3 heterojunctions with enhanced visible light photocatalytic desulfurization under mild conditions. *Journal of Materials Science: Materials in Electronics*, **31**,5622-5634 (2020).

2. Zhou, K., Ding, Y., Zhang, L., Wu, H. and Guo, J.. Synthesis of mesoporous ZnO/TiO2–SiO2 composite material and its application in photocatalytic adsorption desulfurization without the addition of an extra oxidant. *Dalton Transactions*, **49**(5)1600-1612 (2020).

3. Khalafi, T., Buazar, F. and Ghanemi, K.,. Phycosynthesis and enhanced photocatalytic activity of zinc oxide nanoparticles toward organosulfur pollutants. *Scientific reports*, **9**(1), 6866 (2019).

4. Shahbazkhany, S., Salehi, M., Salarvand, Z. and Mousavi-Kamazani, M.,. Photocatalytic oxidative desulfurization of dibenzothiophene solution and real sample of fuel by using Mn-doped ZnO under visible irradiation. *Petroleum Science and Technology*, **41**(19),1811-1830 (2023).

5. Ahmad, M., Yousaf, M., Cai, W. and Zhao, Z.P. Formulation of heterometallic ZIF-8@ Cu/Ni/ZnO@ CNTs heterostructure photocatalyst for Ultra-Deep desulphurization of coal and fuels. *Chemical Engineering Journal*, **453**,139846 (2023).

6. Raeisi, A., Chermahini, A.N. and Momeni, M.M.A novel photocatalytic and photoelectrocatalytic system for oxidative desulfurization of model fuel using BiVO4@ HKUST-1 composite in powder and deposited on fluorine-doped tin oxide. *Journal of Photochemistry and Photobiology A: Chemistry*,**433**,114190 (2022).

7. Xiao-Ming, G.A.O., Jing, W., Feng, F. and Wen-Hong, L.I.,. The Preparation of Cu-BiVO4 and Its Photocatalytic Properties for Desulfurization of Model Oil. *China Petroleum Processing & Petrochemical Technology*, **14**(4),17 (2012).
